# Supplementary material for: Cryo-EM structure of the rhodopsin-Gαi-βγ complex reveals binding of the rhodopsin C-terminal tail to the gβ subunit
Source: eLife. 2019 Jun 28;8:e46041. doi: 10.7554/eLife.46041 (PMC6629373; doi:10.7554/eLife.46041)
Supplement: Supplementary file 1. — Supplementary Table 1. Cryo-EM data collection and refinement statistics. Supplementary Table 2. Crystallographic data and structural refinement of Fab16. [file elife-46041-supp1.docx]

Supplementary tables

|  | **Dataset #1**  **(-) Fab** | **Dataset #2**  **(+) Fab** | **Dataset #3**  **(+) Fab** |
| --- | --- | --- | --- |
| Direct Electron Detector | Falcon III (EC mode) | Falcon III (EC mode) | K2 Summit + GIF (super resolution mode) |
| N. of movies after cleaning | 600 | 600 | 3200 |
| Accelerating Voltage (kV) | 300 | 300 | 300 |
| Total Exposure (e/Å^2^) | 50 | 50 | 60 |
| Number of frames per movie | 50 | 50 | 40 |
| Defocus Range (µm) | -1.5  to -2.5 | -1.5  to -2.5 | -1.5  to -2.5 |
| Automated EM data acquisition software | EPU | EPU | SerialEM |
| Pixel Size (Å/px) | 1.12 | 1.12 | 0.83 |
| Symmetry | C1 | C1 | C1 |
| N. of particles in final map | 69000 | 65000 | 115000 |
| Final Map Resolution (Å) | 15.5 | 6 | 4.38 |
| Map Sharpening B-factor (Å^2^) | -- | -380 | -175 |

**Supplementary Table 1.** Cryo-EM data collection and refinement statistics.

| Data collection | |
| --- | --- |
| Space Group | C 1 2 1 |
| Cell dimensions *a, b, c* (Å)  *α, β, γ* (°) | 169.94, 69.08, 133.76  90.0, 127.2, 90.0 |
| Wavelength (Å) | 1.0 |
| Resolution (Å) | 47.54 – 1.90 (2.05 – 1.90) |
| *R_pim_* | 0.019 (0.457) |
| *R_merge_* | 0.086 (2.121) |
| *I/σI* | 19.8 (1.4) |
| Completeness (%) | 95.1 (86.0) |
| Multiplicity | 20.8 (22.3) |
| CC1/2 | 1.000 (0.701) |

| **Refinement statistics** | |
| --- | --- |
| Refinement program | PHENIX 1.13_2998 |
| Resolution | 47.56 – 1.90 (1.968 – 1.9000) |
| No. Reflections | 67762 (1294) |
| R_work_/R_free_ (%) | 17.95 (36.11)/21.22 (36.11) |
| Number of atoms  Total  Protein  Tetraethylene glycol (PG4)  1,2-ethanediol (EDO)  Triethylene glycol (PGE)  Malic ion (MLT)  Water | 7313  6771  39  48  20  36  390 |
| Ramachandran favoured (%) | 98.27 |
| Ramachandran allowed (%) | 1.73 |
| Ramachandran outliers (%) | 0.00 |
| R.m.s.d Bond legth ($Å$) | 0.005 |
| R.m.s.d Bond angles (°) | 1.14 |
| Averaged B factor (Å^2^)  Protein  Non-water solvent  Water | 50.13  80.09  52.23 |

**Supplementary Table 2.** Crystallographic data and structural refinement of Fab16.
